# Acceptable deviation of labial tubercle and anterior tooth midlines relative to facial midline in smile aesthetics: A retrospective observational study

**DOI:** 10.1097/MD.0000000000030983

**Published:** 2022-10-14

**Authors:** Xiuhong Wang, Jinlie Long, Mei Mei, Jin Huang, Yuan Chen, Yuanzhong Zhou, Jiangtao Zhang

**Affiliations:** a Department of Orthodontics, The Affiliated Stomatology Hospital of Zunyi Medical University, Zunyi Medical University, Zunyi, Guizhou, China; b Department of Laboratory Medicine, Affiliated Hospital of Zunyi Medical University, Zunyi, Guizhou, China; c School of Laboratory Medicine, Zunyi Medical University, Zunyi, Guizhou, China; d School of Public Health, Zunyi Medical University, Zunyi, Guizhou, China.

**Keywords:** acceptable deviation, anterior tooth midline, facial midline, labial tubercle midline, smile aesthetics

## Abstract

Smile aesthetics are mainly influenced by the relative position of facial midline (FC-line), anterior tooth midline (AT-line) and labial tubercle midline (LT-line). However, the acceptable deviation of LT-line and AT-line relative to FC-line is unknown. This study aims to fill the critical gap. We adopted the method of cross-sectional study, the frontal full-face smile photographs of 1 subject set (1 male) were enrolled. Taking the FC-line as the center line, twelve images with 1-mm increment relative to FC-line (right or left deviation, the maximum deviation distance was 3-mm) in LT-line deviation model or LT + AT-line deviation model (LT-line coincided with AT-line basing on LT-line deviation model) were produced. Basing on Q-sort assessment, the images were evaluated by 160 dentists, 165 orthodontic patients and 163 freshmen. And the collected Q-sort scores were subjected to nonparametric comparative analysis using SPSS 18.0. There were significant differences in Q-sort scores among different groups (*P* < .01). When the deviation distance was 1 mm in dentist and orthodontic patient or 2 mm in freshman group, there was no significant difference in smile attractiveness scores between the LT line deviation model and the LT + AT line deviation model (*P* > .05). We also found that the score of male dentist significantly higher than female dentist (*P* < .05). However, the scores of right deviation in dentists and orthodontic patients were significantly lower than those in left deviation (*P* < .05). The acceptable deviation of LT-line and AT-line relative to FC-line should be kept within 2 mm. Besides, raters’ occupation and gender, and deviation direction of model may influence the smile aesthetics.

## 1. Introduction

A smile is one of the most important facial expressions, expressing happiness, friendliness and greetings,^[1]^ as well as appreciation and approval.^[2]^ Hence, it helps to build a trusting relationship between different people.^[3]^

An aesthetic smile is made of various factors.^[4]^ Lip is one of the important smile aesthetics subunits, and previous studies have shown that lip may affect the smile aesthetics. One study in Saudi Arabia investigated the relationship between asymmetry of lip and smile attractiveness, and found that lip asymmetry was negatively correlated with smile attractiveness.^[5]^ Another study found that lips appear to contribute to the aesthetic appeal of a smile in United Kingdom.^[6]^ Moreover, Orestes et al revealed that upper lip distance is closely related to smile attractiveness.^[7]^ Labial tubercle is a part of the lips. Therefore, the influence of labial tubercle midline (LT-line) on smile aesthetics should not be ignored; Similarly, the teeth are an integral part of smile aesthetics.^[8]^ For example, its location affects smile attractiveness.^[9,10]^ Furthermore, the display and size of teeth can also affect the smile aesthetics.^[11,12]^ In addition, facial midline (FC-line) is the sign of facial symmetry. Therefore, the importance of FC-line to smile aesthetics is self-evident. In totally, the LT-line, anterior tooth midline (AT-line) and FC-line are important for smile aesthetic. However, the acceptable deviation of the 2 former relative to the last one in smile aesthetic is unknown.

Smile aesthetics may be influenced by different occupations of participants. A report from Brazil showed that orthodontists and laypeople had different perception on the asymmetries in gingival margins of maxillary canine.^[13]^ Another study shows that compared to dentists in Saudi Arabia, laypeople have higher aesthetic scores for changing smile characteristics.^[14]^ Moreover, in Mexico, the researchers found that there were differences in the perception of smile aesthetics between dental specialists and patients.^[15]^ Thus, in order to avoid the impact of above-mentioned factors, this study recruited participants from 3 different occupations, including orthodontist, orthodontic patient and freshmen.

The Q-sort methodology, originally proposed by William Stephenson,^[16]^ uses progressive forced selection to filter samples and scores subjects on an aesthetic scale from “most unsatisfactory” to “most satisfactory.”^[17]^ The advantages of Q-sort are as follows:^[18]^ First, it can provide plentiful and correlative information that is easy to analyze; Secondly, the Q-information is not logically dependent on essential documents; Finally, the results acquired by analyzing the Q-data are substantial and important significance. Thus, our study was conducted using Q-sort methodology.

When the AT line coincides with the FC line, the aesthetic smile is enhanced,^[19]^ and the LT-line coincide with the above-mentioned 2 lines will make smile more beautiful. Therefore, the deviation of the 3 lines could affect the smile aesthetic. However, acceptable deviation of LT-line and AT-line relative to FC-line in adult has not been investigated. In the current cross-section study, we will explore the acceptable deviation of LT-line and AT-line relative to FC-line in adult smile aesthetics to provide a theoretical basis for orthodontic treatment.

## 2. Materials and Methods

### 2.1. Model selection

One man signed informed consent before being admitting in our study, and the research protocol was approved by the Ethics Committee of Affiliated Stomatological Hospital of Zunyi Medical University. The model had to meet the following criteria: First, there was no abnormal facial soft tissue and history of trauma and maxillofacial surgery; Furthermore, the face was approximately symmetry, the relationship between lips and teeth was harmonious when smiling; Thirdly, the maxillary dentition was complete and orderly arranged, the midline of dental arch was in the middle, there was no scattered space, the gingival and periodontal were healthy, and the labial tubercle stood out.

### 2.2. Questionnaire

The process of questionnaire survey as following: Consultation: We consulted several orthodontics experts after forming the first version of the questionnaire; Pilot survey: In order to accurately convey the purpose of the survey and improve the respondents’ understanding of the questionnaire, the first version of the questionnaire was modified and pre-investigation in a small range; Investigator training: Special training was organized for 4 investigators in advance, and demonstration was conducted in pre-investigation; Questionnaire survey: The questionnaire survey was completed face-to-face to ensure the accuracy of information, which including: gender, occupation and age, etc; Quality control: The questionnaire with issues such as noncompliance with requirements, poor quality of completion, and vacancies were promptly fed back to the respondents, and the respondents were re-invited to fill in the questionnaire.

### 2.3. Creation of the image sets and variables

The model photograph was taken by using the digital camera (NIKOND 5300, Tokyo, Japan) with an exposure time of 1/160 seconds, aperture value of f/5.6 and an ISO value of 320,105-mm macro lens. Taking the FC-line as the center line, the male image was digitally modified with 1-mm increment relative to FC-line (right or left deviation) in LT-line deviation model or LT + AT-line deviation model (LT-line coincided with AT-line basing on LT-line deviation model) by the same professional digital staff using Adobe Photoshop CS6 for a maximum deviation distance of 3-mm. Therefore, 12 digital images were generated by the above-mentioned procedures. At the same time, 7 primary variables were generated, including 0.00, 1.00, 1.00+, 2.00, 2.00+, 3.00, and 3.00+. The meaning of each variable as following: 0.00, original image; 1, the deviation distance between LT-line and FC-line was 1 mm; 1+, basing on variable 1, LT-line coincided with AT-line; 2, the deviation distance between LT-line and FC-line was 2 mm; 2+, basing on variable 2, LT-line coincided with AT-line; 3, the deviation distance between LT-line and FC-line was 3mm; 3+, basing on variable 3, LT-line coincided with AT-line. In addition, gender of volunteers, deviation direction and Q-score were used as secondary variables.

### 2.4. Volunteer inclusion

This study adopts block design, divided all volunteers into different groups according to their different occupations, and conducted a baseline survey in the form of questionnaires. Finally, a total of 160 dentists (78 males, 82 females), 165 orthodontic patients (82 males, 83 females) and 163 freshmen (78 males, 85 females) from June 2015 to February 2017 women) willing to participate in this study. All the participants had to meet the following criterions: Agree to participate in the cooperation and voluntarily provide personal information. No communication barrier, independent thinking ability, and able to understand the content of the questionnaire. For dentists who had 2 or more years of clinical work experience, orthodontic patients who have received orthodontic treatment for more than 6 months, and freshmen who had never undergo orthodontic treatment so far.

### 2.5. Performance of q-sort assessment

Thirteen images were evaluated by each participant, the most (Q-score = 1) and least (Q-score = 9) attractive images were picked in male and female, respectively. Then, the next favorable (Q-score = 2) and unfavorable (Q-score = 8) image were also selected from the remaining pool of 12 images in male and female, respectively. Repeated the above steps until 1 image remains (Q-score = 5), which was called “neutral” image, which means neutral facial attractiveness.

### 2.6. Data analysis

The data were analyzed and processed by IBM SPSS Statistical 18.0 statistical software. The data type of variables is numeric, and the median and interquartile range (IQR) are used for descriptive analysis. Paired Wilcoxon rank sum test was used to analyze the difference of scores between different variables in same group. The differences in scores for the same variable between different rater groups were analyzed by Welch’s ANOVA (group = 3) or Mann–Whitney *U* test (group = 2) after normality test. Test level was set at both sides α = 0.05, *P* < .05 was considered statistically significant.

## 3. Results

The demographic distribution of the rater groups is shown in Table 1. The occupations of rater in this study including 160 dentists, 165 orthodontic patients and 163 freshmen, with an average age of 30.8, 19.4, and 19.0 years, respectively. In order to eliminate the influence of gender on the research results, the gender ratio of each group was close to 1:1, and there were 82 females and 78 males in the dentist scoring group; In patient group, there were 83 females and 82 males; In freshman group, there were 84 females and 79 males. In addition, the male model was 21 years old and was a junior at Zunyi Medical University.

**Table 1:** Demographic distributions of the rater groups.

Variable	Dentist	Patient	Freshman
Number	160	165	163
Age (yrs)	30.8 ± 6.8	19.4 ± 4.1	19.0 ± 1.1
Gender (F/M)	82/78	83/82	84/79

**Note:** Mean ± standard deviation (SD) was used to describe the age.

F = female, M = male.

Table 2 showed the medians, IQRs for smile attractiveness score from male image in 3 rater groups. In dentist group, the higher the deviation distance, the higher the score. However, for patient and freshman groups, the score changes did not depend on the deviation distance. There were significant differences in scores among different groups (*P* < .01), except for the LT-line deviation model which the deviation distance was 2 mm. In the dentist and orthodontic patient groups, there were also significant differences (*P* < .01) in the scores for 2 specific variables. However, when the deviation distance was 1 mm, there was no significant difference between the scores of the LT line deviation and the LT + AT line deviation model (*P* > .05). For freshman, we also found significant differences in scores for 2 specific variables (*P* < .01). However, when the bias distance was 1 mm, the Q-sort scores between the LT-line deviation model and the original image were not significantly different (*P* > .05). In addition, when the deviation distance was 2 mm, the scores of the LT line deviation and the LT + AT line deviation model were also not significantly different (*P* > .05).

**Table 2:** The medians, IQRs for smile attractiveness score from male image in 3 rater groups.

Variable	Dentist			Patient			Freshman			*P* ^**^
	MD	IQR	*P* ^*^	MD	IQR	*P* ^*^	MD	IQR	*P* ^*^	
0.00	1.00	1.00-3.00		3.00	2.00-4.00		4.00	2.00-7.00		.00
1.00	3.00	2.00-4.00	.00^†^	3.00	3.00-5.00	.00^†^	4.00	3.00-6.00	.29^†^	.00
1.00+	3.00	2.00-4.00	.98^‡^	4.00	2.00-5.00	.53^‡^	3.00	2.00-4.00	.00^‡^	.00
2.00	5.00	4.00-6.00	.00^§^	6.00	4.00-7.00	.00^§^	5.00	3.00-7.00	.00^§^	.32
2.00+	6.00	4.00-6.00	.007∥	5.00	4.00-6.00	.04∥	5.00	3.00-7.00	.71∥	.006
3.00	7.00	6.00-7.00	.00^¶^	6.00	6.00-7.00	.00^¶^	6.00	4.00-7.00	.00^¶^	.00
3.00+	8.00	8.00-9.00	.00^#^	8.00	6.00-9.00	.00^#^	7.00	5.00-8.00	.00^#^	.00

**Note:** The difference of score between different variables in same group analyzed by paired Wilcoxon rank sum test; Analysis for the score difference in same variable among the different rater groups by Welch’s ANOVA. Bold value indicates no significant score difference of variables for the intra-group or inter-group.

Abbreviation: 0.00, original image; 1, the deviation distance between labial tubercle midline and facial midline was 1 mm; 1+, basing on variable 1, labial tubercle midline coincided with anterior tooth midline; 2, the deviation distance between labial tubercle midline and facial midline was 2 mm; 2+, basing on variable 2, labial tubercle midline coincided with anterior tooth midline; 3, the deviation distance between labial tubercle midline and facial midline was 3 mm; 3+, basing on variable 3, labial tubercle midline coincided with anterior tooth midline; MD, median; IQR, interquartile range.

*The score difference of different variables in each rater group.

†Comparison for the score difference between 0.00 and 1.00.

‡Comparison for the score difference between 1.00 and 1.00+.

§Comparison for the score difference between 1.00 + and 2.00.

∥ Comparison for the score difference between 2.00 and 2.00+.

¶Comparison for the score difference between 2.00 + and 3.00.

#Comparison for the score difference between 3.00 and 3.00+.

**The score difference in same variable among dentist, patient and freshman rater group.

In Table 3, we presented the medians, IQRs for smile attractiveness score from male image in 3 rater groups after stratification by gender. In dentist, the higher the deviation distance, the higher the score. We also found the score in male dentist was higher than the female dentist when the deviation distance was 1mm in LT + AT-line deviation model (*P* < .05). For orthodontic patient and freshman, there was no significant difference in scores between male and female groups (*P* > .05).

**Table 3:** The medians, IQRs for smile attractiveness score from male image in 3 rater groups after stratification by gender.

Variable	Dentist					Patient					Freshman				
	Male		Female			Male		Female			Male		Female		
	MD	IQR	MD	IQR	*P* ^*^	MD	IQR	MD	IQR	*P* ^*^	MD	IQR	MD	IQR	*P* ^*^
0	1.00	1.00-3.00	1.00	1.00-3.00	.35	3.00	2.00-4.00	3.00	2.00-4.00	.57	5.00	2.00-7.00	4.00	2.00-6.00	.10
1	3.00	2.00-4.00	3.00	2.00-4.00	.95	3.00	3.00-5.00	3.00	2.00-4.00	.20	4.00	3.00-6.00	4.00	3.00-7.00	.67
1+	3.00	3.00-4.00	3.00	3.00-4.00	.02	3.00	2.00-5.00	4.00	2.00-5.00	0.71	3.00	2.00-4.00	3.00	2.00-4.00	.92
2	5.00	4.00-6.00	5.00	4.00-6.00	.24	6.00	4.00-7.00	6.00	4.00-7.00	.47	5.00	3.00-6.00	5.50	3.50-7.00	.21
2+	6.00	4.00-6.00	6.00	4.00-6.00	.68	5.00	4.00-6.00	5.00	4.00-7.00	.98	6.00	3.00-7.00	5.00	3.00-7.00	.27
3	7.00	6.00-7.00	7.00	6.00-7.00	.51	6.00	6.00-7.00	6.00	5.00-7.00	.23	6.00	4.00-7.00	6.00	4.00-7.00	.16
3+	8.00	8.00-9.00	8.00	8.00-9.00	.35	8.00	6.00-8.50	8.00	6.00-9.00	.08	7.00	4.00-8.00	7.00	5.00-8.00	.68

**Note:** The score difference in same variable between male and female subgroup analyzed by Mann–Whitney U test. Bold value indicates no significant score difference of variables for the intra-group or inter-group.

Abbreviation: 0.00, original image; 1, the deviation distance between labial tubercle midline and facial midline was 1 mm; 1+, basing on variable 1, labial tubercle midline coincided with anterior tooth midline; 2, the deviation distance between labial tubercle midline and facial midline was 2 mm; 2+, basing on variable 2, labial tubercle midline coincided with anterior tooth midline; 3, the deviation distance between labial tubercle midline and facial midline was 3 mm; 3+, basing on variable 3, labial tubercle midline coincided with anterior tooth midline; MD, median; IQR, interquartile range.

*The score difference in same variable between male and female subgroup.

The medians, IQRs for smile attractiveness score from male image in 3 rater groups after stratification by deviation direction were shown in Table 4. For LT + AT-line deviation model, right deviation score was lower than left deviation score in orthodontic patient when the deviation distance was 1 mm (*P* < .01). When the deviation distance was 2 mm, right deviation score was also lower than left deviation score in dentist and orthodontic patient (*P* < .01). In addition, when the deviation distance was 3 mm, right deviation score was lower than left deviation score in dentist and orthodontic patient (*P* < .05); In LT-line deviation model, the right deviation score was also lower than left deviation in dentist when the deviation distance was 3 mm (*P* < .01).

**Table 4:** The medians, IQRs for smile attractiveness score from male image in 3 rater groups after stratification by deviation direction.

Variable	Dentist					Patient					Freshman				
	Right		Left			Right		Left			Right		Left		
	MD	IQR	MD	IQR	*P* ^*^	MD	IQR	MD	IQR	*P* ^*^	MD	IQR	MD	IQR	*P* ^*^
1	3.00	2.00-4.00	3.00	3.00-3.00	.10	4.00	3.00-5.00	3.00	2.00-5.00	.27	4.00	3.00-6.00	4.00	3.00-6.00	.75
1+	3.00	2.00-4.00	3.00	3.00-4.00	.15	3.00	1.00-4.00	4.00	3.00-6.00	.00	3.00	2.00-4.00	3.00	3.00-4.00	.17
2	5.00	4.00-6.00	5.00	4.00-6.00	1.00	5.00	4.00-7.00	6.00	4.00-7.00	.56	5.00	3.00-6.00	5.00	3.00-7.00	.92
2+	5.00	4.00-6.00	6.00	6.00-6.00	.00	4.00	4.00-6.00	6.00	4.00-7.00	.00	5.00	3.00-7.00	6.00	3.00-7.00	.41
3	7.00	6.00-7.00	7.00	7.00-7.00	.00	7.00	4.00-7.00	6.00	6.00-7.00	.59	6.00	4.00-7.00	6.00	4.00-7.00	.75
3+	8.00	8.00-9.00	9.00	8.00-9.00	.02	7.00	6.00-8.00	8.00	7.00-9.00	.00	7.00	5.00-8.00	7.00	5.00-9.00	.60

**Note:** The score difference in same variable between right and left deviation direction subgroup analyzed by Mann–Whitney U test. Bold value indicates no significant score difference of variables for the intra-group or inter-group.

Abbreviation: 1, the deviation distance between labial tubercle midline and facial midline was 1 mm; 1+, basing on variable 1, labial tubercle midline coincided with anterior tooth midline; 2, the deviation distance between labial tubercle midline and facial midline was 2 mm; 2+, basing on variable 2, labial tubercle midline coincided with anterior tooth midline; 3, the deviation distance between labial tubercle midline and facial midline was 3 mm; 3+, basing on variable 3, labial tubercle midline coincided with anterior tooth midline; MD, median; IQR, interquartile range.

*The score difference in same variable between right and left deviation direction subgroup.

## 4. Discussion

This study assessed the perceptions of smile aesthetics among different participants, including orthodontists, orthodontic patients, and freshmen, and explored acceptable deviation of the LT-line and AT-line relative to the FC-line in smile aesthetics. We found that the acceptable deviation of LT-line and AT-line relative to FC-line should be kept within 2 mm. Moreover, the occupation and gender of raters, and deviation direction of model may affect the smile aesthetics.

This study proposed that people in different occupations have different perceptions of facial aesthetic evaluation, which was similar to previous research. Similar to our findings, Talic et al assessed and compared the perceived attractiveness scores of altered smile features between laypeople and dentists, and they found that the former scored higher than the latter.^[14]^ Furthermore, Correa et al showed the orthodontists were more sensitivity to the maxillary alterations than laypeople while comparing the perception of asymmetries in the gingival margins of maxillary canine between the 2 groups.^[13]^ In addition, Beyer et al explored the impact of tooth midline position on smile aesthetics and showed that orthodontists and dentists were more likely than patients to detect midline deviation.^[20]^

In this study, we also revealed that the acceptable deviation of LT-line and AT-line relative to FC-line should be kept within 2 mm, and when the LT-line deviation reached 3 mm, people are willing to accept that the AT-line coincide with the FC-line, which was coincident with available research findings. A study from America enrolled 120 persons, including orthodontists, general dentists, orthodontic patients and patients’ parents, demonstrated that 2.2 ± 1.5 mm was an acceptable dental midline deviation for the volunteers when evaluating altered image.^[20]^ The other study enrolled 20 orthodontists and 20 young adult layperson for scoring the altered image and showed that the layperson might give a higher attractiveness score while the discrepancy between the dental and FC-lines was 2 mm.^[21]^ Furthermore, one study from Israel, the researcher presented 3 types photograph sets (the deviation distance of AT-line <1 mm, 1-2 mm and >2 mm, respectively) to 5 dentists and 5 nondental personnel. The results showed that nearly half of the participations were unable to detect the AT-line deviation of <2 mm, but this investigation did not analyze the different perception between dentists and nondental personnel.^[19]^ However, Kokich et al explored the perceptions of different occupation participants with respect to minor variations in smile aesthetics, they found lay people were unable to detect the AT-line deviation until reached 3 or 4-mm.^[22]^ In addition, Pinho et al also investigated the effect of AT line deviation on smile aesthetics in volunteers of different occupations, and the results showed that for nonprofessionals, the midline deviation became perceptible when it was equal to or greater than 3.0 mm.^[23]^

After stratification by gender, we found that the score in male dentist was higher than the female dentist when the deviation distance was 1 mm in LT + AT-line deviation model, suggesting that male rater dentists were more tolerant of deviation. Therefore, the gender of the rater may affect the smile aesthetics. There have been no studies on smile aesthetics using the LT + AT line bias model, nor a gender-stratified analysis based on such studies. However, in other research models, such as the FC line deviation model, male raters have a higher perception of the deviance threshold than females.^[24]^ For AT-line deviation model, one study demonstrated that both male and female rater were more tolerant of AT-line deviation in male subjects than in female subjects, but female raters were more tolerant of AT line bias in male subjects higher than male raters;^[25]^ Another study showed that the maximum acceptable deviation of AT lines was higher in males than in females (3 mm vs 2 mm).^[26]^ Therefore, the gender of the rater may be an important factor affecting smile aesthetics.

After stratification by deviation direction, the right deviation score was lower than left deviation score in dentist and orthodontic patient when the deviation distance >1 mm, indicating that the right deviation was more preferred than left deviation. At present, there is no research on smile aesthetics using the LT + AT line deviation model, and there is no stratified analysis of the deviation direction based on such research. However, in other research models, for example, in the AT-line bias model, the rater’s preference does not depend on the direction of the AT-line bias.^[25]^ Therefore, further research is needed to investigate whether the deviated directions of the LT and AT lines affect smile aesthetics.

One strength of our study was that we recruited participants from many different occupations, which allowed us to explore the acceptable deviation of LT-line and AT-line relative to FC-line in smile aesthetics, and to compare the effect of different occupations on smile aesthetics. Moreover, since the gender of the participants may influence smile aesthetics,^[27,28]^ we therefore analyzed the different scores of smile attractiveness after stratification by gender in this study. Thirdly, we analyzed different score of smile attractiveness after stratification by deviation direction. Finally, face-to-face interviews were conducted with all participants to provide information and ensure reliability.

However, some limitations of this study cannot be neglected. Firstly, there is no internationally accepted standard photo for assessing smile aesthetics. Therefore, the comparison of our findings with other studies was not entirely objective. Secondly, only 3 occupational groups were included in our study, and other occupational groups also shared their perceptions of smile aesthetics. Thirdly, only the male image was included in this study, however, there may be different smile attractiveness between female and male images according to previous study.^[29]^ Fourthly, non-significant results may be easily affected by the sample size, gender, occupation, deviation direction and so on. Therefore, we advocate that the above factors should be considered when analyzing non-significant results. Last but not least, participants at different times may have different perception of smile aesthetics, which may also influence smile aesthetics. In view of the above limitations, further studies should focus on the acceptable deviation of LT-line and AT-line relative to FC-line in smile aesthetics.

## 5. Conclusion

In conclusion, the acceptable deviation of LT-line and AT-line relative to FC-line should be kept within 2 mm. Besides, raters’ occupation and gender, and deviation direction of model may influence the smile aesthetics.

## Author contributions

**Conceptualization:** Jiangtao Zhang, Yuanzhong Zhou, Jinlie Long, Xiuhong Wang.

**Data curation:** Jinlie Long, Xiuhong Wang.

**Formal analysis:** Jinlie Long, Xiuhong Wang.

**Project administration:** Xiuhong Wang, Mei Mei, Jin Huang, Yuan Chen.

**Supervision:** Jiangtao Zhang, Yuanzhong Zhou.

**Software:** Jinlie Long.

**Writing – original draft:** Jinlie Long.

**Writing – review & editing:** Jinlie Long, Jiangtao Zhang, Yuanzhong Zhou.
